# Spindle orientation: What if it goes wrong?

**DOI:** 10.1016/j.semcdb.2014.06.014

**Published:** 2014-10

**Authors:** Dan T. Bergstralh, Daniel St Johnston

**Affiliations:** a The Gurdon Institute, Tennis Court Road, Cambridge CB2 1QN, United Kingdom; b The Department of Genetics, University of Cambridge, Tennis Court Road, Cambridge CB2 1QN, United Kingdom

**Keywords:** Spindle orientation, Metaphase, NuMa/Mud/LIN-5, Tumorigenesis, Epithelia, Neuroblast

## Abstract

The angle of cell division is critical in at least two contexts. It can determine cell fate, as it does in developing neural tissue. It can also dictate tissue architecture, as it does in many epithelia. One way to ensure the correct angle of cell division is through controlled orientation of the spindle at metaphase. What happens when that control is lost? Ongoing work suggests that the consequence of metaphase spindle misorientation may be significant, but multiple mechanisms exist to protect the cell and the tissue. We speculate that one such mechanism involves a recently identified anaphase activity for two of the key players at metaphase: NuMA (Mud, LIN-5) and dynein.

## Highlights

• Spindle orientation is important to both cell fate and tissue architecture.
• Multiple mechanisms act to limit the consequences of metaphase spindle misorientation.
• One such mechanism may involve the anaphase function of NuMA/Mud/Lin-5.

## 1 Introduction

Evidence from multiple organisms demonstrates that the angle of division is central to cell fate in neural tissues and to the formation of epithelia [1]. A complex machinery exists to orient the metaphase spindle in a variety of cell types and organisms [2]. In this review we will consider the consequence of its failure.

As defects in both cell fate and tissue organization are implicated in tumor development, one possibility that must be considered is that spindle misorientation contributes to cancer. This suggestion has been widely discussed in the literature – reviews include (but are not limited to) [3], [4], [5]. The article by Pease and Tirnauer in 2011 provides an excellent account of work up until that time, with particular attention to mammalian carcinomas. We will pick up from there, with further attention given to recent advances in vertebrate models and to evidence accumulating in non-mammalian systems.

Our interpretation of this evidence suggests that in most contexts the angle of division is too important to be entrusted to metaphase spindle orientation alone. The organism relies on multiple mechanisms to protect itself from misoriented divisions and ensure the integrity of tissues as they develop.

### 1.1 How are spindles oriented at metaphase?

Work in *Caenorhabditis elegans*, *Drosophila*, and cultured mammalian cells has identified a canonical spindle orientation machinery that operates during metaphase. This machinery exerts a pulling force between factors localized at the cell cortex and astral microtubules, and thereby pulls indirectly on spindle poles to bring them into orientation [6]. While the list of core factors is slowly expanding, at least four appear to be necessary in most, if not all, contexts. In *Drosophila* they are called Gαi (GOA-1 and GPA-16 in *C. elegans*), Pins (LGN or GPSM2 in vertebrates, GPR1/2 in *C. elegans*), Mud (vertebrate NuMA, LIN-5 in *C. elegans*), and dynein/dynactin.

**Fig. 1:**
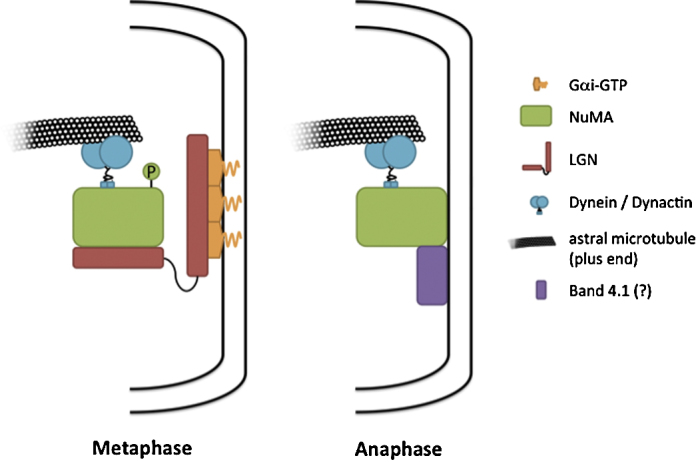
NuMA/Mud and dynein/dynactin exert pulling from the cortex at both metaphase and anaphase. During metaphase, NuMA is maintained at the cortex by LGN, which is in turn anchored by Gαi. Phosphorylation by CDK1 (not shown) prevents it from localizing at the cortex without LGN. At anaphase NuMA is dephosphorylated and can bind to the cortex independently of LGN. This may or may not occur through interaction with Band 4.1 protein(s).

The identification of these molecules and their functions has been reviewed elsewhere (recently in [7], [8]). A brief overview of the complex follows: The G-protein subunit Gαi, which is myristoylated, binds to the plasma membrane. This may depend on the guanine exchange factor Ric-8, though its role in different tissues is not yet clear. Gαi-GDP serves as a cortical anchor for Pins, binding to its C-terminal GoLoco motifs. Pins in turn serves as a dock for Mud, to which it binds *via* N-terminal tetricopeptide repeats. Mud binds to the dynein/dynactin complex, which provides the minus-end directed motor activity that generates the pulling force (Fig. 1).

The pathway just described explains how cortical proteins can affect spindle orientation. Recent work also suggests that spindle orientation information may originate from the metaphase plate. In HeLa cells, a chromosome-derived gradient of Ran-GTP feeds back to the cortex to locally inhibit association of LGN and NuMA with the membrane. They are thus concentrated at sites more proximal to the spindle poles [9]. The functional consequence of this activity is not yet known. It may serve to reinforce and maintain spindle alignment once it has been achieved. Another possibility is that it helps promote division along the long axis of the cell, since the chromosomes are farthest from the cortex in this orientation.

Much of our understanding of spindle orientation, as in the case just described, derives from work done in cultured cells. What happens to a single cell when the spindle fails to orient at metaphase?

### 1.2 A new role for NuMA

The majority of attention given to spindle orientation to date is centered on machinery that operates at metaphase, but the activity of the spindle at this point is only a warm-up for the main event: the segregation of chromosomes. Recent work from four groups demonstrates that elements of the metaphase machinery, namely NuMA and dynein, have an additional role at anaphase [10], [11], [12], [13]. The results of these studies are consistent with the following three points: (1) During anaphase, NuMA and dynein localize to two cortical crescents at opposite sides of the cell, along the axis of division. (2) This localization depends on the activity of Cdk1, which is thought to phosphorylate NuMA to restrict its localization prior to anaphase. (3) Anaphase localization of NuMA is independent of LGN and Gαi (Fig. 1).

The studies differ in their details however, and raise several questions.

Firstly, how is NuMA anchored to the cortex during anaphase? It may involve the cytoskeletal protein Band 4.1 [10], [11]. In HeLa cells, Band 4.1 and Band 4.1-like 2 provide an anaphase-specific mechanism for localizing NuMA independently of LGN [11]. In mouse keratinocytes, however, the cortical localization of NuMA at anaphase occurs even if both its Band 4.1-binding region and LGN-binding regions are removed [10]. In Cos 7 cells, NuMA associates directly with the lipid membrane during anaphase *via* a newly recognized membrane binding domain [12]. This does not rule out a role for Band 4.1, but suggests at least that it does not provide an anchor.

Secondly, what is the function of NuMA and dynein during anaphase? One attractive possibility is that it may be to help ensure symmetric cell division, in respect to daughter cell size and/or DNA content. Defects in either are associated with tissue disorganization and cancer [14], [15].

If the cleavage furrow is not at the center of the cell, there is a risk that cytoplasm and/or chromosomes may be split unevenly during division. Thus unequal chromosome segregation and size asymmetry might be predicted if division occurs along the incorrect axis. The possibility that spindle misorientation promotes these asymmetries can be tested in HeLa cells, which take on a triangular shape when cultured on an L-shaped fibronectin micropattern. In accordance with Hertwig's rule, they divide along their long axis, which is the hypotenuse of the triangle. In the absence of LGN or Gαi, the rule may be disobeyed; neither NuMA nor dynein are recruited to the cortex at metaphase and spindle orientation is randomized [11]. However, cell division in HeLa cells is reliably symmetric regardless of the division axis.

This may be because NuMA and dynein act after metaphase – independently of LGN – to ensure that the spindle is *centered* in the cell even if division is occurring at an incorrect angle [11]. Dynein-dependent centering has been previously illustrated in metaphase-arrested HeLa cells, in which the spindle oscillates relative to the cortex such that neither spindle pole stays too close to the cortex [9]. Kiyomitsu and Cheeseman have now shown that centering continues through anaphase, at which point it is sometimes achieved through asymmetric expansion of the plasma membrane [11]. If one side of the membrane is too close to a spindle pole, the membrane will expand to move away from it. Thus the distance from each pole to the membrane is equalized. In order to work, this mechanism requires the spindle pole at the side that does not expand to stay in place. If that pole is not anchored (presumably by a pulling force generated by localized NuMA and dynein), the spindle moves toward the expanding membrane and size asymmetry is promoted rather than resolved.

Both defective anaphase spindle anchoring and daughter cell asymmetry are observed in cells depleted of LGN, Band 4.1 and Band 4.1-like 2, even if the cells are not plated on an L-pattern ([11] and I. Cheeseman, personal communication). These findings support a model in which Band 4.1 proteins anchor Mud at anaphase to ensure spindle centering.

Data from another cell type complicates the picture. Using mouse keratinocytes, Seldin et al. observed that mechanical stretching of the substrate promotes metaphase spindle orientation along the stretched (long) axis, and this effect depends on the Band 4.1-binding domain of NuMA [10]. Since this domain is dispensable for anaphase localization of NuMA in these cells, this result suggests that Band 4.1 acts during metaphase to promote division along the long axis [10]. While these findings indicate that the relevant activity of Band 4.1 is at metaphase, they do not contradict a role for NuMA and dynein in spindle centering during anaphase.

However, another anaphase-specific activity for NuMA and dynein has also been proposed. Rather than provide spatial cues that protect daughter cell size, NuMA and dynein might act at anaphase to help pull apart the spindle poles [12], [13]. In support of this view, it has been observed that the distance between spindle poles during anaphase is shorter in NuMA depleted HeLa cells [13]. A pulling activity at anaphase might be expected as a normal feature of cell division. Might it also help safeguard against the consequence of a misoriented spindle? Given that NuMA and dynein are on opposite sides of the division plane, pulling on both spindle poles at anaphase should help to ensure that the two daughters have an equal DNA complement, if not an equal size, even if the spindle is not along the long axis. We note that mis-segregation can promote aneuploidy, itself a hallmark of cancer.

Future studies must be relied on to clarify the activity of NuMA and dynein at anaphase. It may be noted however that both functions proposed so far might provide protection from the deleterious effects of misoriented metaphase spindles to the cell. The consequence of misorientation however must also be considered in respect to the tissue.

We also note that this is not the only instance of an LGN (Pins)-independent role for NuMA (Mud). In *Drosophila*, the four classical alleles of *mud* are all associated with reduced viability and female sterility, which is caused by spindle defects during meiosis II [16], [17]. These defects are not observed in *pins* mutants; the transheterozygous allele combination *pins*^*p62*^*/pins*^*p89*^ is associated with reduced viability, but adult flies are healthy and can lay fertilized eggs [18]. *mud* and *pins* mutants also have different phenotypes in the developing brain, as will be discussed below.

## 2 Spindle orientation and cell fate in neural tissues

In both the fly and the chick, division orientation plays a critical role in populating the brain with neurons. Both systems rely on neural progenitor cells, which divide asymmetrically to produce two distinct cell types. One of the daughters continues to self-renew, whereas the other becomes a differentiating cell. Neural progenitor cells in these systems are subtly distinguished from stem cells in that they are not immortal. Rather than divide indefinitely, they are programmed to disappear once neural development is complete.

There are thus at least two defects that can lead to excessive neurons. The first of these is a failure in cell programming, such that the progenitor cell fails to stop dividing. A role for spindle orientation in this process is illustrated in the chick embryo, in which neural progenitor cells divide in a pseudostratified neuroepithelium. Directed division orientation maintains progenitors in the apical part of the tissue, called the ventricular zone, while their post-mitotic, differentiating daughters move basally and accumulate in the mantle zone. Disruption of LGN in this tissue allows a progenitor to move into the mantle zone, where it can continue to divide for up to four days – even if the ventricular zone progenitors have stopped [19]. It must be noted that while hyperplasia in the chick brain reflects the importance of spindle orientation to tissue architecture, the proliferation of ectopic progenitor cells in the chick brain is not unlimited. Furthermore, this phenomenon is not observed in other vertebrates. The loss of LGN function in the neuroepithelium of the developing mouse also causes movement of progenitors out of the ventricular zone, but these progenitors have the same proliferative life-span as the appropriately localized cells [20].

The second defect that may be implicated in neuron overpopulation is a failure of differentiation. This is illustrated in the *Drosophila* brain, a workhorse for the study of spindle orientation over the past two decades (reviews include [4], [21], [22], [23]). *Drosophila* neural progenitor cells, called neuroblasts, divide to produce a self-renewing neuroblast and a differentiating cell, typically a ganglion mother cell (GMC), which ultimately gives rise to neurons. This process continues until pupariation, at which point development is complete. Excess neurons in the larva can result from a failure in daughter cell fate such that neither of the two daughter cells becomes a GMC and both continue to self-renew.

The fate of the two daughter cells of a neuroblast is decided by protein determinants that are unequally distributed between them. Among the cell fate determinants distributed to the GMC are Prospero and Brat (Brain tumor), so named because it behaves as a tumor suppressor in the brain [24], [25]. These proteins act to repress neuroblast cell fate and promote GMC differentiation. In their absence both daughters continue to divide as neuroblasts, leading to overgrowth of the tissue [26], [27], [28], [29]. The relevance of this phenomenon to cancer is demonstrated by elegant tissue transplantation experiments. When transferred into the abdomens of adult hosts, *brat* or *pros* mutant larval brain tissue can develop into large masses with characteristics of malignant tumors, including metastasis [30]. These masses are derived from neuroblasts. Remarkably, they can be propagated indefinitely by subsequent rounds of re-transplantation [30].

How is the asymmetric distribution of cell fate factors to the two daughters achieved? Prior to division, the cell fate determinants segregate to two sides of the cell – one apical and one basal. Segregation relies on cortical polarity, perhaps most importantly on the apical kinase aPKC and the basolateral factor Lethal (2) giant larvae, which have an antagonistic relationship that is governed at the onset of mitosis by Aurora A [27], [31], [32], [33]. The overgrowth of *lethal giant larvae* mutant neuroblasts in the larva is the first instance of tumorigenesis characterized in the fly, and results from a failure in daughter cell differentiation [34], [35], [36].

To achieve asymmetric distribution, the neuroblast orients its spindle along the apical–basal axis so that one daughter (the neuroblast) will inherit the apical factors and the other (the GMC) the basal ones. This orientation relies on the canonical spindle machinery in cooperation with an additional factor called Inscuteable, which localizes Pins (Partner of Inscuteable) along the apical cell cortex and thereby ensures that one spindle pole is drawn apically [18], [37], [38]. The mammalian ortholog of Inscuteable, called mInsc, also plays an important role in division orientation of neural progenitor cells [39], [40], [41].

These results demonstrate the importance of cell fate decisions to tissue organization in the brain. Since those decisions rely on the asymmetric distribution of determinants, which is driven by oriented cell division, one would expect defective neuroblast spindle orientation to be catastrophic to the tissue. The best evidence to support this possibility comes from observations made in *mud* mutant flies. The adult brains of *mud* mutants are characterized by morphological defects in memory-associated regions called mushroom bodies [42]. Mushroom bodies are comprised of neurons called Kenyon cells, which are generated by four neuroblasts during development. Both the number of these neuroblasts and the number of Kenyon cells is increased in *mud* mutants [43], [44]. This observation can be explained by defective spindle orientation; neuroblasts that divide perpendicular to the apical–basal axis segregate their apical cell fate determinants symmetrically [45]. Somewhat surprisingly, the basal cell fate determinant Miranda is typically segregated asymmetrically even if the neuroblast divides perpendicular to the apical–basal axis [45]. However, this is not sufficient to change cell fate; the inheritance of apical factors will confer neuroblast identity even if the daughter cell also receives basal factors [45]. Equal distribution of apical fate determinates to *mud* mutant daughter cells thus allows the production of two self-renewing cells rather than one [45]. In turn, these extra neuroblasts go on to produce more Kenyon cells than in the wild type fly.

These results strongly implicate spindle misorientation in tissue overgrowth, but two caveats are raised. Firstly, tissue defects are primarily associated with the mushroom body of *mud* mutant flies, but supernumerary neuroblasts are also observed elsewhere in *mud* mutant brains [43], [44], [45]. This suggests that either the mushroom body is particularly susceptible to overgrowth or that some of the morphological defect is attributable to another function of Mud. Secondly, Mud is thought to act as an effector of Inscuteable and Pins, but mushroom body overgrowth in *inscuteable* or *pins* mutant flies has not been reported. The number of larval neuroblasts is decreased, rather than increased, in *pins* mutant brains [46]. Furthermore, the orientation of neuroblast division and distribution of cell fate factors in these mutants are usually recovered after metaphase, a phenomenon called “telophase rescue” [35], [36], [46], [47].

Why do not *inscuteable* and *pins* mutant neuroblasts overproliferate? One potential explanation is that unlike Mud, they regulate cortical polarity as well as spindle orientation. Inscuteable and Pins are each required to localize the important apical determinant aPKC at metaphase [46], [48]. aPKC is implicated in neuroblast self-renewal, and its exclusively apical localization may be important for its activity [45], [46], [48]. Thus the failure to keep aPKC localized only at the apical cortex may limit proliferation. This is an attractive proposal, but the picture is complicated by tissue-transplantation evidence. As with *pros* and *brat*, malignant neuroblastomas develop following transplantation of neural tissue from *pins* mutant larvae into adults [30]. The absence of Pins function can cause tissue overgrowth after all, but only if the tissue is isolated from its normal environment.

Some inferences may be drawn from these observations. Firstly, mechanisms are in place to promote asymmetric distribution of basal factors to neuroblast daughters regardless of spindle orientation. This underlines an important point; cells may have more than one way of getting around a problem. Secondly, a cue (or cues) acts to limit the proliferation of *pins* mutant neuroblasts in the larval brain. This cue likely originates outside the brain, as neuroblasts fail to respond when the neural tissue is in the abdomen. Whether or not such a cue acts on *inscuteable* mutant neuroblasts has not been tested, but it is established that these neuroblasts do not expand *in vivo* despite randomized metaphase spindle orientation. Thirdly, since *mud* mutant neuroblasts can multiply in the brain we can conclude Mud is functionally distinct from the other metaphase spindle orientation factors examined.

Given the results recently reported in mammalian cells (and discussed above), it is tempting to propose that the distinct activity for Mud is carried out at anaphase, during the correction that occurs in *inscuteable* and *pins* mutant neuroblasts. It must be noted, however, that an anaphase role for Mud has not yet been established outside of mammalian cultured cells.

Finally, we note again that *pins* and *mud* mutant flies are outwardly healthy. To date the only tissue defects reported for *mud* are in the brain, and these reflect cell fate discrepancies after neuroblast misdivision, which is in the fly a developmental problem. Both Pins and Mud are involved in spindle orientation in symmetrically dividing epithelial cells, but there is no evidence yet for tissue disorganization [49], [50], [51].

## 3 Spindle orientation in epithelia

Spindle orientation in epithelia is not linked to cell fate; epithelial cells divide symmetrically to produce identical daughters. Division orientation is nonetheless vital, as it can determine tissue architecture. Within an epithelial layer, division tends to occur along the plane of the tissue such that the layer expands and is maintained. The direction of tissue expansion may be determined by the planar orientation of division, as in the developing mouse lung. During the morphogenesis of epithelial tubes, divisions occur along the length of the lumen [52]. This ensures that the growth in lumen length outpaces its growth in circumference, such that tubes are long and thin [52].

In epithelia that consist of multiple layers, stratification is initiated by divisions that are perpendicular, rather than parallel, to the plane of the tissue; these divisions are part of the normal development of both the mouse epidermis and the terminal end buds of mammary ducts [53], [54], [55]. The loss of spindle control might thus be expected to change the number of layers. Extraneous perpendicular divisions could be a step toward cancer; in a superfluous layer a cell may be isolated from its normal signaling environment and thus the cues that govern its proliferation.

Somewhat surprisingly, knockdown of either NuMA or Pins using lentiviral shRNA in the embryonic mouse epidermis *decreases* both stratification and differentiation, resulting in a thinner and more permeable tissue [54]. The architecture of the tissue is disrupted, but tumors are not observed. This suggests the possibility that in the absence of spindle control, divisions are parallel by default. Alternatively, perpendicular divisions alone are insufficient to promote stratification. This possibility has been investigated in *Drosophila.*

### 3.1 *Drosophila* tissues

While most of the attention given to spindle orientation in flies has been on neuroblasts, several studies have also considered spindle orientation in epithelial cells. Metaphase spindles in these tissues tend to orient along the plane of the tissue, perpendicular to the apical–basal axis [49], [56], [57], [58]. The evidence to date indicates that *Drosophila* epithelial cells utilize the canonical orientation machinery, which localizes along the lateral cortex and cooperates with at least one more protein, the lateral polarity factor Discs large (Guilgur et al., 2012; Bergstralh et al., 2013b; Y.-I. Nakajima et al., 2013) [49], [50], [51].

In two epithelial tissues, namely the embryonic ectoderm and the larval optic lobe, it appears that this machinery can be co-opted by exogenously expressed Inscuteable, which localizes apically and causes spindles to reorient [37], [59]. Disorganization of the tissue is not observed in either case [37], [59]. These observations suggest that epithelial tissues are protected from the potentially tumorigenic consequence of misoriented divisions. One explanation is that the incorrectly divided cells undergo apoptosis. In at least one *Drosophila* epithelium, the imaginal wing disc of the larva, this is the case.

The wing disc is comprised of a thick, single layer of pseudo-stratified epithelial cells. Cell division occurs along the plane of the apical surface following apical-directed migration of the nucleus from the middle of the tissue, a process called interkinetic nuclear migration. In addition to Mud and Discs large, spindle orientation in these cells involves atypical protein kinase C (aPKC), the lateral polarity factor Scribble, and several proteins that regulate the strength of the acto-myosin cortex [49], [51]. aPKC, Dlg, Scribble, and acto-myosin regulators all have functions outside of the spindle, but Mud is implicated only in spindle regulation. To date, RNAi-mediated knockdown of Mud in the disc is the strongest experiment testing the consequence of spindle misorientation to *Drosophila* epithelia. In this tissue the disruption of Mud allows for division to occur obliquely relative to the plane of the tissue [60]. This in turn promotes basal cell extrusion and apoptosis [51]. Extrusion and apoptosis are also observed following disruption of aPKC and any of the other factors mentioned [49], [51]. The disc is thus protected from whatever consequence a misplaced daughter cell has to the tissue.

The mechanism whereby basal extrusion is initiated remains unclear. A misoriented division results in one daughter positioned apically, as in the wild type, and one daughter positioned more basally. In and of itself, this mispositioning would not seem likely to pose a substantial problem; cell bodies drop back toward the center of the tissue after division anyway. Rather, it may be that planar orientation allows for even distribution of apically positioned structural components to the two daughters, and a failure to inherit these factors leads to cell death.

In the wing disc, misoriented divisions cause cell death. What happens when the apoptoic program is defective, as may be the case in a transformed cell? Exogenous expression of the baculovirus protein p35 is used in *Drosophila* to protect cells from apoptosis. In a wing disc with misoriented spindles, expression of p35 gives rise to basal tumor-like masses with mesenchymal characteristics [49], [51]. These results are consistent with a two-hit hypothesis whereby spindle misorientation may contribute to tumorigenesis but another mutation is required, in this case to protect the cell from death.

Because cell division can be studied in the context of a tissue – both *in* and *ex vivo – Drosophila* has proven a useful model for examining spindle orientation and the consequence of its failure. But cancer is really a problem for animals with longer lifespans, including us.

### 3.2 Vertebrate models

The Madin-Darby Canine Kidney (MDCK) cell line is a long-established model system for the study of epithelial cell apical–basal polarity. Since the advent of three-dimensional culture they have also been used as a model for tissue organization. MDCK cells cultured in a gel can divide to form cysts – spherical sheets of epithelial cells surrounding fluid-filled lumens – that might be considered epithelial acini. As expected, spindles tend to orient in parallel to the plane of the tissue, and this orientation requires LGN [61], [62]. A role for NuMA has not been determined, but spindle misorientation is also observed in cysts stably expressing Inscuteable, which suggests that it can hijack the spindle orientation machinery, as it does in *Drosophila* epithelia [63]. MDCK cysts have also provided insight into the workings of canonical machinery; spindle misorientation is observed in cysts expressing the LGN point mutant S401A, which does not localize exclusively along the lateral cell cortex at metaphase [64]. Phosphorylation at this residue is thus critical to its localization and function.

Cysts have also been used to identify new spindle orientation factors. 3D cultured Caco-2 cells can also form cysts, and these have been used to demonstrate a role for the small GTPase Cdc42 in spindle orientation [65]. Subsequent studies using MDCK cysts showed that this effect relies on the Cdc42 guanine exchange factors Tuba and Intersectin 2 [62], [66]. Cdc42 is an activator of the protein kinase aPKC, already mentioned as an apical polarity factor [67], [68]. Chemical inhibition of aPKC also causes spindle misorientation in MDCK cysts [66]. This is in turn because aPKC phosphorylates LGN at S401 in these cells, although it should be mentioned that this interaction may not be conserved between species [50], [69], [70].

More recent work in MDCK cysts has identified a role for the scaffold protein IQGAP1, which binds the EGF receptor at the basal surface, in spindle orientation. RNAi-mediated depletion of IQGAP1 leads to spindle misorientation, as does treatment with EGF [71]. Although the relationship between these proteins and the canonical spindle machinery remains unclear, it has been observed that mitotic NuMA localization is affected in siRNA IQGAP1 cells. Pins localization however remains normal [71].

The consequence of spindle misorientation to cyst formation is drastic. Disruption of any of the spindle orientation factors described leads to a tissue disorganization phenotype characterized by multiple lumens. These results underline the importance of spindle orientation in determining tissue architecture and show that a mispositioned cell can have dramatic consequences. However, it should be noted that in these experiments spindle orientation is incorrect from the outset of cystogenesis; in other words, prior to the formation of an epithelial-like structure. These results are almost certainly relevant to tissue architecture, but they may not provide support for a model in which spindle misorientation promotes cancer.

## 4 Conclusions

The orientation of the metaphase spindle is under tight control in a variety of cell types, a fact that underlines its importance. As we have discussed, the loss of control may affect cell fate and tissue architecture. Are the consequences even more drastic? The evidence to date suggests that metaphase spindle orientation may potentiate disorganization and allow for overgrowth in oncogenic circumstances, but is unlikely to cause tumorigenesis on its own. We suggest that the orientation of division is so important that it relies on multiple pathways. NuMA and dynein, for example, may provide a second orientation at anaphase. Additional work will help to clarify this point. It may also be that most tissues have mechanisms in place to minimize the consequence of misorientation, such as apoptosis in the wing disc. We expect that other mechanisms will be uncovered in the future.

## References

[1] Williams S.E., Fuchs E. (2013). Oriented divisions, fate decisions. Curr Opin Cell Biol.

[2] Lu M.S., Johnston C.A. (2013). Molecular pathways regulating mitotic spindle orientation in animal cells. Development.

[3] Gonzalez C. (2007). Spindle orientation, asymmetric division and tumour suppression in *Drosophila* stem cells. Nat Rev Genet.

[4] Knoblich J.A. (2010). Asymmetric cell division: recent developments and their implications for tumour biology. Nat Rev Mol Cell Biol.

[5] Pease J.C., Tirnauer J.S. (2011). Mitotic spindle misorientation in cancer—out of alignment and into the fire. J Cell Sci.

[6] Kotak S., Busso C., Gönczy P. (2012). Cortical dynein is critical for proper spindle positioning in human cells. J Cell Biol.

[7] Bergstralh D.T., Haack T., St Johnston D. (2013). Epithelial polarity and spindle orientation: intersecting pathways. Philos Trans R Soc Lond Ser B Biol Sci.

[8] Kotak S., Gönczy P. (2013). Mechanisms of spindle positioning: cortical force generators in the limelight. Curr Opin Cell Biol.

[9] Kiyomitsu T., Cheeseman I.M. (2012). Chromosome- and spindle-pole-derived signals generate an intrinsic code for spindle position and orientation. Nat Cell Biol.

[10] Seldin L., Poulson N.D., Foote H.P., Lechler T. (2013). NuMA localization, stability, and function in spindle orientation involve 4.1 and Cdk1 interactions. Mol Biol Cell.

[11] Kiyomitsu T., Cheeseman I.M. (2013). Cortical dynein and asymmetric membrane elongation coordinately position the spindle in anaphase. Cell.

[12] Zheng Z., Wan Q., Meixiong G., Du Q. (2014). Cell cycle-regulated membrane binding of NuMA contributes to efficient anaphase chromosome separation. Mol Biol Cell.

[13] Kotak S., Busso C., Gönczy P. (2013). NuMA phosphorylation by CDK1 couples mitotic progression with cortical dynein function. EMBO J.

[14] Siegel J.J., Amon A. (2012). New insights into the troubles of aneuploidy. Ann Rev Cell Dev Biol.

[15] Lloyd A.C. (2013). The regulation of cell size. Cell.

[16] de Belle J.S., Heisenberg M. (1996). Expression of *Drosophila* mushroom body mutations in alternative genetic backgrounds: a case study of the mushroom body miniature gene (mbm). Proc Natl Acad Sci U S A.

[17] Yu J.X., Guan Z., Nash H.A. (2006). The mushroom body defect gene product is an essential component of the meiosis II spindle apparatus in *Drosophila* oocytes. Genetics.

[18] Yu F., Morin X., Cai Y., Yang X., Chia W. (2000). Analysis of partner of inscuteable, a novel player of Drosophila asymmetric divisions, reveals two distinct steps in inscuteable apical localization. Cell.

[19] Morin X., Jaouen F., Durbec P. (2007). Control of planar divisions by the G-protein regulator LGN maintains progenitors in the chick neuroepithelium. Nat Neurosci.

[20] Konno D., Shioi G., Shitamukai A., Mori A., Kiyonari H., Miyata T. (2007). Neuroepithelial progenitors undergo LGN-dependent planar divisions to maintain self-renewability during mammalian neurogenesis. Nat Cell Biol.

[21] Januschke J., Gonzalez C. (2008). Drosophila asymmetric division, polarity and cancer. Oncogene.

[22] Morin X., Bellaïche Y. (2011). Mitotic spindle orientation in asymmetric and symmetric cell divisions during animal development. Dev Cell.

[23] Siller K.H., Doe C.Q. (2009). Spindle orientation during asymmetric cell division. Nat Cell Biol.

[24] Wright T.R. (1987). The genetic and molecular organization of the dense cluster of functionally related, vital genes in the DOPA decarboxylase region of the *Drosophila melanogaster* genome. Results Probl Cell Differ.

[25] Chu-LaGraff Q., Wright D.M., McNeil L.K., Doe C.Q. (1991). The prospero gene encodes a divergent homeodomain protein that controls neuronal identity in Drosophila. Development.

[26] Bello B., Reichert H., Hirth F. (2006). The brain tumor gene negatively regulates neural progenitor cell proliferation in the larval central brain of *Drosophila*. Development.

[27] Betschinger J., Mechtler K., Knoblich J.A. (2006). Asymmetric segregation of the tumor suppressor brat regulates self-renewal in *Drosophila* neural stem cells. Cell.

[28] Choksi S.P., Southall T.D., Bossing T., Edoff K., de Wit E., Fischer B.E. (2006). Prospero acts as a binary switch between self-renewal and differentiation in Drosophila neural stem cells. Dev Cell.

[29] Lee C.-Y., Wilkinson B.D., Siegrist S.E., Wharton R.P., Doe C.Q. (2006). Brat is a Miranda cargo protein that promotes neuronal differentiation and inhibits neuroblast self-renewal. Dev Cell.

[30] Caussinus E., Gonzalez C. (2005). Induction of tumor growth by altered stem-cell asymmetric division in *Drosophila melanogaster*. Nat Genet.

[31] Rolls M.M., Albertson R., Shih H.-P., Lee C.-Y., Doe C.Q. (2003). Drosophila aPKC regulates cell polarity and cell proliferation in neuroblasts and epithelia. Journal of Cell Biology.

[32] Plant P.J., Fawcett J.P., Lin D.C.C., Holdorf A.D., Binns K., Kulkarni S. (2003). A polarity complex of mPar-6 and atypical PKC binds, phosphorylates and regulates mammalian Lgl. Nat Cell Biol.

[33] Wirtz-Peitz F., Nishimura T., Knoblich J.A. (2008). Linking cell cycle to asymmetric division: aurora-A phosphorylates the par complex to regulate Numb localization. Cell.

[34] Gateff E. (1978). Malignant neoplasms of genetic origin in Drosophila melanogaster. Science.

[35] Peng C.Y., Manning L., Albertson R., Doe C.Q. (2000). The tumour-suppressor genes lgl and dlg regulate basal protein targeting in Drosophila neuroblasts. Nature.

[36] Ohshiro T., Yagami T., Zhang C., Matsuzaki F. (2000). Role of cortical tumour-suppressor proteins in asymmetric division of Drosophila neuroblast. Nature.

[37] Kraut R., Chia W., Jan L.Y., Jan Y.N., Knoblich J.A. (1996). Role of inscuteable in orienting asymmetric cell divisions in Drosophila. Nature.

[38] Schaefer M., Shevchenko A., Shevchenko A., Knoblich J.A. (2000). A protein complex containing Inscuteable and the Galpha-binding protein Pins orients asymmetric cell divisions in Drosophila. Curr Biol.

[39] Zigman M., Cayouette M., Charalambous C., Schleiffer A., Hoeller O., Dunican D. (2005). Mammalian inscuteable regulates spindle orientation and cell fate in the developing retina. Neuron.

[40] Postiglione M.P., Jüschke C., Xie Y., Haas G.A., Charalambous C., Knoblich J.A. (2011). Mouse inscuteable induces apical-basal spindle orientation to facilitate intermediate progenitor generation in the developing neocortex. Neuron.

[41] Lancaster M.A., Knoblich J.A. (2012). Spindle orientation in mammalian cerebral cortical development. Curr Opin Neurobiol.

[42] Technau G., Heisenberg M. (1982). Neural reorganization during metamorphosis of the corpora pedunculata in *Drosophila melanogaster*. Nature.

[43] Prokop A., Technau G.M. (1994). Normal function of the mushroom body defect gene of *Drosophila* is required for the regulation of the number and proliferation of neuroblasts. Dev Biol.

[44] Bowman S.K., Neumüller R.A., Novatchkova M., Du Q., Knoblich J.A. (2006). The Drosophila NuMA Homolog Mud regulates spindle orientation in asymmetric cell division. Dev Cell.

[45] Cabernard C., Doe C.Q. (2009). Apical/basal spindle orientation is required for neuroblast homeostasis and neuronal differentiation in *Drosophila*. Dev Cell.

[46] Lee C.-Y., Robinson K.J., Doe C.Q. (2006). Lgl, Pins and aPKC regulate neuroblast self-renewal versus differentiation. Nature.

[47] Schober M., Schaefer M., Knoblich J.A. (1999). Bazooka recruits Inscuteable to orient asymmetric cell divisions in Drosophila neuroblasts. Nature.

[48] Wodarz A., Ramrath A., Grimm A., Knust E. (2000). Drosophila atypical protein kinase C associates with Bazooka and controls polarity of epithelia and neuroblasts. Journal of Cell Biology.

[49] Guilgur L.G., Prudencio P., Ferreira T., Pimenta-Marques A.R., Martinho R.G. (2012). Drosophila aPKC is required for mitotic spindle orientation during symmetric division of epithelial cells. Development.

[50] Bergstralh D.T., Lovegrove H.E., St Johnston D. (2013). Discs large links spindle orientation to apical–basal polarity in *Drosophila* epithelia. Curr Biol.

[51] Nakajima Y.-I., Meyer E.J., Kroesen A., McKinney S.A., Gibson M.C. (2013). Epithelial junctions maintain tissue architecture by directing planar spindle orientation. Nature.

[52] Tang N., Marshall W.F., McMahon M., Metzger R.J., Martin G.R. (2011). Control of mitotic spindle angle by the RAS-regulated ERK1/2 pathway determines lung tube shape. Science.

[53] Lechler T., Fuchs E. (2005). Asymmetric cell divisions promote stratification and differentiation of mammalian skin. Nature.

[54] Williams S.E., Beronja S., Pasolli H.A., Fuchs E. (2011). Asymmetric cell divisions promote Notch-dependent epidermal differentiation. Nature.

[55] Huebner R.J., Lechler T., Ewald A.J. (2014). Developmental stratification of the mammary epithelium occurs through symmetry-breaking vertical divisions of apically positioned luminal cells. Development.

[56] Egger B., Chell J.M., Brand A.H. (2008). Insights into neural stem cell biology from flies. Philos Trans R Soc Lond Ser B Biol Sci.

[57] Foe V.E. (1989). Mitotic domains reveal early commitment of cells in *Drosophila* embryos. Development.

[58] Fernández-Miñán A., Martín-Bermudo M.D., González-Reyes A. (2007). Integrin signaling regulates spindle orientation in *Drosophila* to preserve the follicular-epithelium monolayer. Curr Biol.

[59] Egger B., Boone J.Q., Stevens N.R., Brand A.H., Doe C.Q. (2007). Regulation of spindle orientation and neural stem cell fate in the Drosophila optic lobe. Neural Dev.

[60] Nakajima H., Tanoue T. (2010). Epithelial cell shape is regulated by Lulu proteins via myosin-II. J Cell Sci.

[61] Zheng Z., Zhu H., Wan Q., Liu J., Xiao Z., Siderovski D.P. (2010). LGN regulates mitotic spindle orientation during epithelial morphogenesis. J Cell Biol.

[62] Rodriguez-Fraticelli A.E., Vergarajauregui S., Eastburn D.J., Datta A., Alonso M.A., Mostov K. (2010). The Cdc42 GEF Intersectin 2 controls mitotic spindle orientation to form the lumen during epithelial morphogenesis. J Cell Biol.

[63] Zhu J., Wen W., Zheng Z., Shang Y., Wei Z., Xiao Z. (2011). LGN/mInsc and LGN/NuMA Complex Structures Suggest Distinct Functions in Asymmetric Cell Division for the Par3/mInsc/LGN and Gαi/LGN/NuMA Pathways. Mol Cell.

[64] Hao Y., Du Q., Chen X., Zheng Z., Balsbaugh J.L., Maitra S. (2010). Par3 controls epithelial spindle orientation by aPKC-mediated phosphorylation of apical Pins. Curr Biol.

[65] Jaffe A.B., Kaji N., Durgan J., Hall A. (2008). Cdc42 controls spindle orientation to position the apical surface during epithelial morphogenesis. J Cell Biol.

[66] Qin Y., Meisen W.H., Hao Y., Macara I.G., Tuba (2010). a Cdc42 GEF, is required for polarized spindle orientation during epithelial cyst formation. J Cell Biol.

[67] Joberty G., Petersen C., Gao L., Macara I.G. (2000). The cell-polarity protein Par6 links Par3 and atypical protein kinase C to Cdc42. Nat Cell Biol.

[68] Lin D., Edwards A.S., Fawcett J.P., Mbamalu G., Scott J.D., Pawson T. (2000). A mammalian PAR-3-PAR-6 complex implicated in Cdc42/Rac1 and aPKC signalling and cell polarity. Nat Cell Biol.

[69] Johnston C.A., Hirono K., Prehoda K.E., Doe C.Q. (2009). Identification of an aurora-A/PinsLINKER/Dlg spindle orientation pathway using induced cell polarity in S2 cells. Cell.

[70] Peyre E., Jaouen F., Saadaoui M., Haren L., Merdes A., Durbec P. (2011). A lateral belt of cortical LGN and NuMA guides mitotic spindle movements and planar division in neuroepithelial cells. J Cell Biol.

[71] Bañón-Rodríguez I., Gálvez-Santisteban M., Vergarajauregui S., Bosch M., Borreguero-Pascual A., Martin-Belmonte F. (2014). EGFR controls IQGAP basolateral membrane localization and mitotic spindle orientation during epithelial morphogenesis. Embo J.

